# Ecological impacts of large-scale disposal of mining waste in the deep sea

**DOI:** 10.1038/srep09985

**Published:** 2015-05-05

**Authors:** David J. Hughes, Tracy M. Shimmield, Kenneth D. Black, John A. Howe

**Affiliations:** 1 Scottish Association for Marine Science, Oban, Argyll PA37 1QA, United Kingdom; 2 SAMS Research Services Ltd. (SRSL), Lismore Suite, Malin House, European Marine Science Park, Oban, Argyll PA37 1SZ, United Kingdom

## Abstract

Deep-Sea Tailings Placement (DSTP) from terrestrial mines is one of several large-scale industrial activities now taking place in the deep sea. The scale and persistence of its impacts on seabed biota are unknown. We sampled around the Lihir and Misima island mines in Papua New Guinea to measure the impacts of ongoing DSTP and assess the state of benthic infaunal communities after its conclusion. At Lihir, where DSTP has operated continuously since 1996, abundance of sediment infauna was substantially reduced across the sampled depth range (800–2020 m), accompanied by changes in higher-taxon community structure, in comparison with unimpacted reference stations. At Misima, where DSTP took place for 15 years, ending in 2004, effects on community composition persisted 3.5 years after its conclusion. Active tailings deposition has severe impacts on deep-sea infaunal communities and these impacts are detectable at a coarse level of taxonomic resolution.

The deep-sea bed, until recently a remote and largely pristine environment, is now subject to growing anthropogenic pressures from industrial-scale resource extraction, accidental pollution and deliberate waste disposal^1^. The huge scale of the 2010 *Deepwater Horizon* oil spill - to date the largest single accidental pollution incident in the deep sea^2^-necessitated a rapid response from the deep-sea research community^3^,^4^, and a need for continuing studies to monitor its long-term consequences. The *Deepwater Horizon* incident also drew unprecedented public attention to the issue of human impacts on the deep sea, since reinforced by coverage of proposals for the commercial mining of deep-sea mineral resources^5^. A much less-publicised impact on the deep sea is the intentional disposal of solid waste (tailings) from land-based mines, a process involving material input greater than that of the *Deepwater Horizon* and continuing for years or decades rather than months. Deep-Sea Tailings Placement (DSTP) involves discharge of finely-ground rock slurry from an outfall below the base of the surface mixed layer, the tailings then flowing as a near-bed density current to depths >1000 m^6^,^7^. The technique builds on experience gained from over a century of tailings disposal in Norwegian coastal fjords, in some cases to water depths of several hundred metres^8^. The environmental risks posed by tailings impoundments and other land-based storage methods^9^ make DSTP an attractive and economic disposal option for developing nations heavily reliant on exploitation of mineral resources. It is currently used in Indonesia, Papua New Guinea (PNG) and on the Turkish Black Sea at mines which meet the necessary conditions of access to deep (>1000 m) water via a steep (>12^0^) continental or island slope, and is being considered as a disposal option at several new or projected mines in south-east Asia and the western Pacific^10^. The practice is highly controversial, with many local communities and non-governmental organisations voicing concerns about potential environmental impacts^11^. Research to date has focused on pelagic biota and nearshore coral communities in the vicinity of DSTP outfalls^12^,^13^,^14^,^15^, but nothing is known of effects at the deep-sea bed which forms the final repository for the bulk of the discharged material. Deep-Sea Tailings Placement entails massive inputs of fine sediment, containing residual heavy metals derived from the terrestrial ore body^16^ (and potentially additional contaminants introduced by chemical processing of the ore) into bathyal environments regarded as hotspots of deep-sea biomass and biodiversity^17^,^18^. The lack of information on its ecological consequences is therefore a significant gap in our knowledge of anthropogenic impacts in the deep sea.

In November 2007, we sampled the sediments around two island mines in eastern PNG (Fig. 1a) to quantify the benthic impacts of ongoing DSTP and assess community states following its conclusion. The Lihir gold mine discharges ~100,000 ML tailings slurry year^−1^ (containing ~2.5 Mt solids) from an outfall at 128 m depth on the east coast of the island^12^,^13^ (Fig. 1b). Mining began in 1996, with a projected duration of 44 years. Tailings consist of 93% silt particulates, with residual particulate and dissolved metals (including zinc, copper, arsenic, cadmium, mercury and lead)^12^ and process chemicals. There are no published data for trace metal content in tailings-affected sediments off Lihir, but dispersal models ground-truthed by sediment sampling indicate a depositional “footprint” extending across a broad plain up to 20 km east of the outfall and to depths of at least 2000 m^19^. The gold/silver mine on Misima Island discharged a total of ~90 Mt tailings from 1989 until the end of operations in May 2004. The outfall was located at 112 m depth on a very steep (>45^0^) submarine slope leading into the 1500 m deep Bwagaoia Basin (Fig. 1c). Seismic profiling indicates a tailings layer tens of metres thick in places and covering approximately 20 km^2^ of the basin floor^20^. Unlike Lihir, the main deposition zone at Misima is confined by seafloor topography but this does not exclude the possibility of wider dispersal by resuspension. No information is available on trace metal content of the tailings discharged at Misima.

There are no reliable pre-DSTP baseline benthic community data for either Lihir or Misima. At Lihir we therefore compared infaunal communities at three benthic stations (depths ~800, 1700, 2000 m) in the tailings “footprint” (L1-L3) with depth-matched reference stations (L4-L6) west of the island (Fig. 1b), in an area previously reported to be clear of tailings^19^. Available bathymetric data for the Misima area were very poor and with no accurate estimate of the total tailings “footprint”, reference stations were more difficult to define *a priori*. We therefore sampled three stations (M1-M3) in the Bwagaoia Basin depocentre along a transect extending from the tailings outfall, and two (M4, M5) separated from the basin by a bathymetric high but potentially exposed to resuspended tailings (Fig. 1c). A sixth station (M6) was separated from the other five by an island chain and was expected to lie outside any likely zone of influence from the mine. The six Misima stations covered a depth range of 1250–1793 m. Location data for all sampling stations are listed in Supplementary Table S1.

## Results

### Lihir

**Figure 2:**
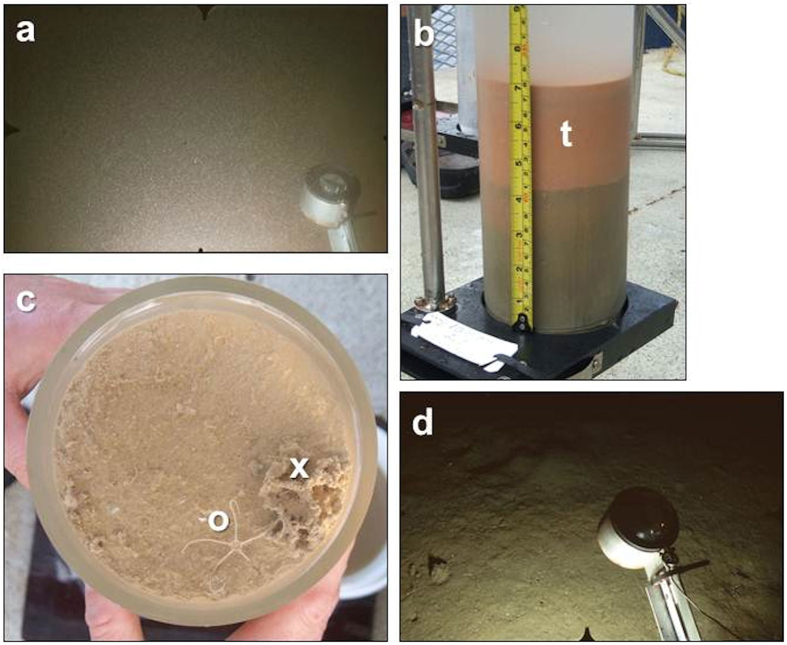
Seabed images and cored sediments from stations around Lihir (**a**) Drop-camera image from L1 (depth 850 m) showing seabed completely obscured by suspended particles. (**b**) Core from L1 with a thick orange layer of semi-fluid, freshly-deposited tailings (t) overlying more consolidated sediment. (**c**) Surface of a core from L5 (depth 1715 m) showing natural sediment with fine-scale biogenic relief, a small ophiuroid (o) and a xenophyophore (x). (**d**) Drop-camera image from L4 (depth 800 m) showing natural sediment seabed with biogenic traces. In this image the compass arm has been forced upward by accidental contact with the seabed. Image width in (**a**) and (**d**) is approximately 1 m across the lower edge. Core diameter in (**b**) and (**c**) is 10 cm.

Cored sediments from the two Lihir station groups were visually and geochemically distinct, with stations east of the island showing unequivocal evidence of tailings deposition. On drop-camera images from L1 the seabed was completely obscured by a dense haze of suspended particles (Fig. 2a). Cores from L1-L3 had a 3–7 cm thick surface layer of watery, fine-grained orange mud, representing freshly-deposited tailings, overlying consolidated muddy sands with thin laminations of coarser material, probably left by intermittent slumps of natural sediment and waste rock from the mine (Fig. 2b). Cores appeared devoid of biological activity. In contrast, drop-camera images from L4 showed a sediment seabed with burrow openings and other biogenic traces (Fig. 2d). Cored sediments at L4-L6 were homogeneous, moderately well-sorted muddy sands with no visible tailings layer. Core surfaces showed abundant small-scale biogenic relief and occasional small epifauna (Fig. 2c). Solid-phase metal inventories differed between stations east and west of the island (Supplementary Table S2). Cores from L1-L3 had lower solid-phase calcium, a marker for natural biogenic sedimentation (inventory to 14 cm depth, 3647–16284 g m^−2^) than cores from L4-L6 (range 16769–22887 g m^−2^). In contrast, the content of several tailings-derived trace metals was much higher in cores from L1-L3 with, for example, solid-phase lead ranging from 3.8–6.5 g m^−2^ at L1-L3 versus 0.6–0.7 g m^−2^ at L4-L6.

**Figure 3:**
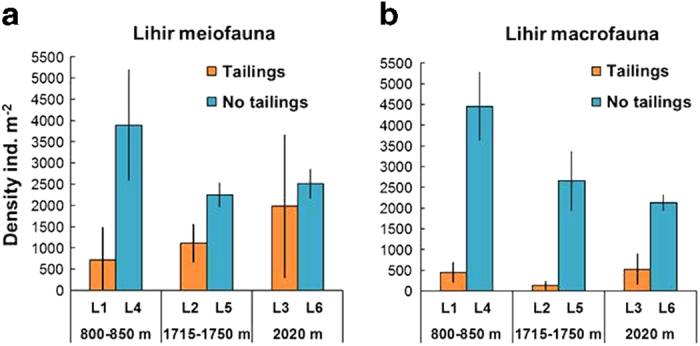
Abundance of metazoans (>250 μm) at stations around Lihir Bars represent means (±SD) of replicate corer drops (n = 3–7 drops station^−1^, see Supplementary Table S1), with densities standardised to individuals m^−2^. Depth-matched tailings and reference (no tailings) stations are shown in adjacent bars.

Mean densities of total metazoan meiofauna were significantly higher (α = 0.05) at reference stations in both the 800–850 m and 1715–1750 m depth zones than at the corresponding stations in the tailings “footprint” (Fig. 3a) (Mann-Whitney U-test L1 ≠ L4, U = 28.0, P = 0.0058; L2 ≠ L5, U = 15.0, P = 0.0358). At 2020 m, numbers at tailings and reference stations were not significantly different (Mann-Whitney U-test L3 ≠ L6, U = 25.0, P = 0.1098). Meiofaunal composition differed consistently across the depth range, with harpacticoid copepods accounting for 70–98% of individuals at L1-L3 versus 40–51% at L4-L6 (Supplementary Table S3). Nematodes showed the converse pattern of higher relative abundance (40–53%) at reference stations. The difference between pooled tailings and reference stations was highly statistically significant (Mann-Whitney U-test for percentage Copepoda, Tailings ≠ Reference, U = 349.5, P = 0.0025).

Total macrofaunal abundance was significantly higher (α = 0.05) at reference stations at all three depth intervals (Fig. 3b) (Mann-Whitney U-test L1 ≠ L4, U = 28.0, P = 0.0058; L2 ≠ L5, U = 15.0, P = 0.0369; L3 ≠ L6, U = 21.0, P = 0.0142). The very sparse macrofauna observed at L1-L3 consisted almost entirely (83–97% individuals) of small polychaetes in the families Cossuridae, Paraonidae and Spionidae. Stations L4-L6 supported a much more diverse macrofauna with a higher-taxon structure typical of bathyal sediments^21^. Polychaetes (17–27 families) accounted for 58–64% individuals, the remainder consisting of bivalves, peracarid crustaceans, echinoderms and various minor groups.

Among benthic Foraminifera (forams) in the >250 μm size fraction, living organic-walled (allogromiid) species were entirely absent in cores from stations L1-L3, but present at all three reference stations (Table 1). Living calcareous forams were absent from L1 and L2, but abundant at the corresponding reference stations (L3, L4). Calcareous foram densities were low but not significantly different at the two deepest stations L3 (tailings) and L6 (reference) (Mann-Whitney U-test L3 ≠ L6, U = 10.5, P = 1.0000).

### Misima

**Figure 4:**
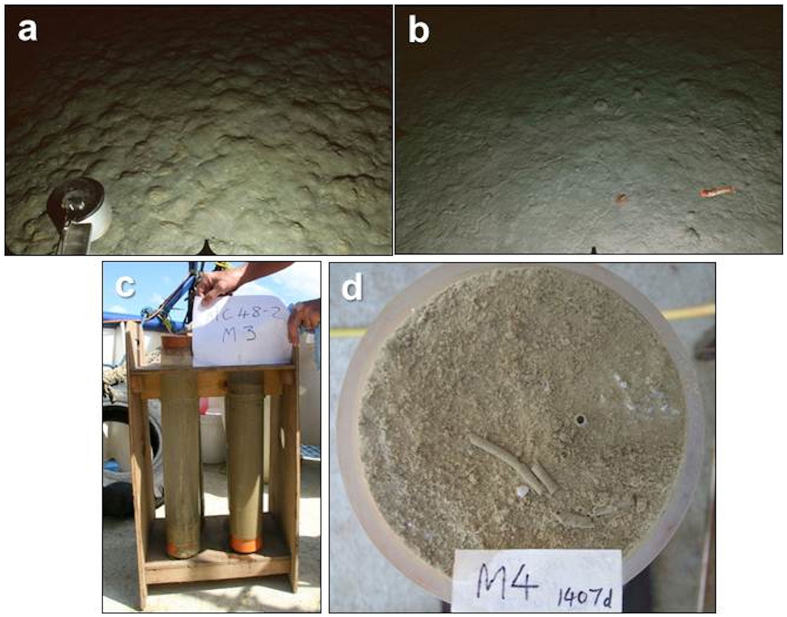
Seabed images and cored sediments from stations around Misima (**a**) Drop-camera image from M1 (depth 1380 m) showing irregular seabed topography with no visible biogenic features. (**b**) Drop-camera image from M5 (depth 1704 m) showing seabed with epifaunal trails, burrow openings and feeding marks. Two decapod crustaceans (swimming shrimp and small galatheid on sediment surface) at lower right. Compass arm not fitted to camera frame at this station. (**c**) Two cores from M3 (depth 1467 m) showing homogeneous brown sediment. (**d**) Surface of a core from M4 (depth 1793 m) showing flocculent superficial material, polychaete tube opening and adjacent tube fragments. Image width in (**a**) and (**b**) is approximately 1 m across the lower edge. Core diameter in (**c**) and (**d**) is 10 cm.

Seabed images from M1 showed an irregular, lumpy bedform suggestive of a recent disturbance event (Fig. 4a). There were no visible epifauna or biogenic traces. In contrast, images from M5 showed a more regular seabed topography with occasional epifauna and abundant biogenic traces including small mounds, burrow openings and trails (Fig. 4b). Cored sediments were similar in appearance at all six Misima stations, with no superficial fresh tailings layer (Fig. 4c, d). However, we found a clear geochemical contrast between stations M1-M3 and M5-M6 (Supplementary Table S4), with the former group showing lower values for solid-phase calcium and higher values for tailings-derived trace metals. Station M4 showed values intermediate between these two groups. Geochemical data therefore indicate high tailings content at M1-M3, a lower level of input at M4, possibly by resuspension from the depocentre, and entirely natural sediments at M5 and M6.

**Figure 5:**
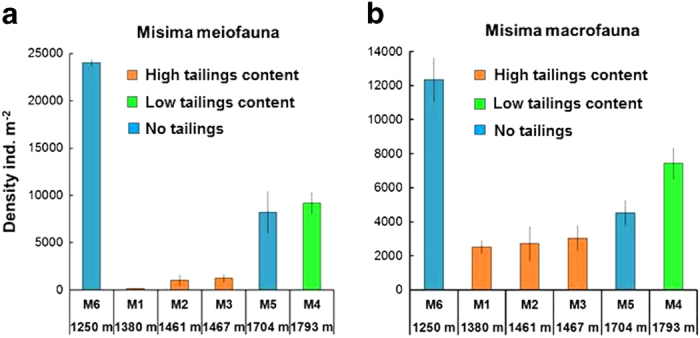
Abundance of metazoans (>250 μm) at stations around Misima Bars represent means (±SD) of replicate corer drops (n = 3 drops station^−1^, except for M3, where n = 6 drops), with densities standardised to individuals m^−2^. Stations are arranged along the X-axis in order of increasing water depth, and colour-coded according to the geochemical evidence for presence of tailings in cored sediments.

Stations M1-M3 showed very low densities of total metazoan meiofauna in comparison with the tailings-free M6 station (Fig. 5a). Values at M4 and M5 showed the expected depth-related decline^22^ relative to M6, but were not significantly different (α = 0.05) from each other (Mann-Whitney U-test M4 ≠ M5, U = 12.0, P = 0.6625). Metazoan meiofaunal density therefore showed no evidence of a tailings effect at M4. Higher-taxon representation at M1 followed the pattern of the Lihir tailings stations, with harpacticoid copepods comprising 71% of the metazoan meiofauna. The copepod percentage declined to 52% at M2 and 38% at M3, while the relative abundance of nematodes increased. Stations M4-M6 grouped together closely in terms of meiofaunal composition, with 21–23% copepods and 72–76% nematodes (Supplementary Table S5). Total macrofaunal abundance was similarly highest at M6, lower at M4 and M5 and uniformly lowest at M1-M3 (Fig. 5b). Macrofaunal abundance at M4 (low tailings content) was not significantly different from the tailings-free station M5 at very similar water depth (Mann-Whitney U-test, U = 6.0, P = 0.0809). Macrofaunal higher-taxon structure at M1 was distinctive (Supplementary Table S6), with similar representation of polychaetes (43% total individuals) and bivalves (36%) and a high abundance (18%) of echiuran worms, a group which is usually a very minor component of the deep-sea macrofauna^21^. Stations M2 and M3 were almost identical in higher-taxon composition with 72–73% polychaetes, 19–20% bivalves and very few (<1%) echiurans. Stations M4-M6 had 54–68% polychaetes, 15–17% bivalves and no echiurans. The Bwagaoia Basin tailings stations (M1-M3) were united by the extreme rarity of peracarid crustaceans (amphipods, isopods and tanaidaceans), which accounted for only 0–2% of the macrofaunal individuals. Outside the basin (M4-M6) peracarids made up 11–24% of the total community, proportions much more typical of natural deep-sea sediments^23^. Metazoan meio- and macrofauna therefore showed reduced densities and anomalous community structure in the main tailings depocentre. No tailings impacts were apparent at M4-M6.

**Figure 6:**
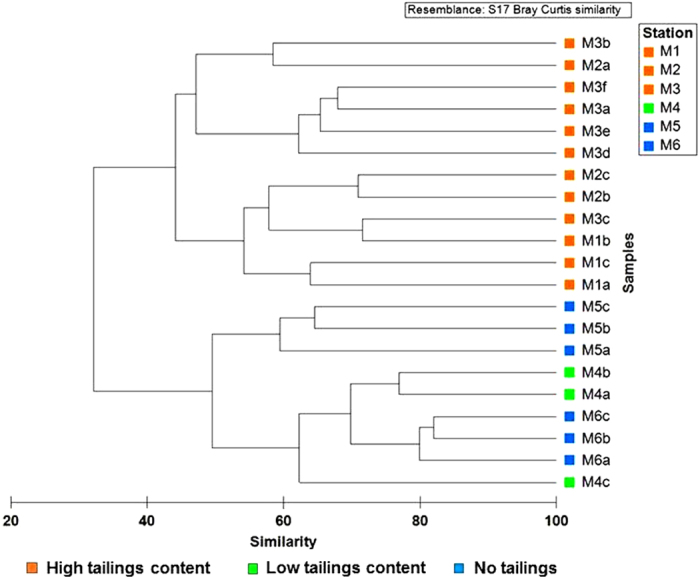
Clustering of Misima samples by polychaete family abundance Bray-Curtis similarity of untransformed polychaete family abundance data. Samples are labelled by station number (M1-M6) with lower-case letters representing individual corer drops. Abundance data for corer drops are standardised to ind. m^−2^ to correct for small differences in the number of individual cores per drop. Samples are colour-coded according to the geochemical evidence for presence of tailings in cored sediments from each station.

Hierarchical cluster analysis of Misima samples by polychaete family abundance also supported a primary division (at ~30% similarity) between stations in the Bwagaoia Basin (M1-M3) and those outside (M4-M6) (Fig. 6). Analysis of Similarity (ANOSIM) showed that the two clusters were significantly different (Global R = 0.797, P = 0.001). Within the basin cluster, stations M1, M2 and M3 were not consistently separated from each other. Samples from M4, where solid-phase metal data indicated some tailings input, grouped with samples from the tailings-free station M6 at >60% similarity. In SIMPER (Primer^TM^ v.6)^24^, the five largest contributors to dissimilarity between the two station groups were the families Spionidae, Paraonidae, Syllidae, Cirratulidae and Lumbrineridae, which collectively accounted for 56% of total dissimilarity. With the exception of Lumbrineridae, the leading families were consistently more abundant in the M4-M6 station group (Supplementary Table S7).

Living organic-walled benthic forams (allogromiids) (>250 μm) were recorded at all six Misima stations. Mean densities were higher at stations outside the tailings depocentre (M4-M6), although with large ranges of variation at all localities (Table 2). Allogromiid densities at the natural-sediment stations combined (M5-M6) were significantly (α = 0.05) greater than at the high-tailings stations in the basin depocentre (M1-M3) (Mann-Whitney U-test, U = 47.0, P = 0.0037). However, as for metazoan meiofauna and macrofauna, allogromiid densities at station M4 were not significantly different from those at M5 (Mann-Whitney U-test, U = 9.0, P = 0.6625). A notable feature was the very high abundance of two calcareous foraminiferan taxa at stations in the Bwagaoia Basin. A porcellanous miliolid, provisionally identified as *Quinqueloculina* sp., occurred at high density at stations M1-M3 but was not recorded elsewhere. A hyaline-walled rotaliid, provisonally identified as *Buliminella* sp., was superabundant at stations M1 and M2, present in very low numbers at M4, and not recorded elsewhere. Additional to these two species the six stations supported a range of other calcareous forams (Table 2).

## Discussion

Impact assessment using a formal “BACI” (Before-After-Control-Impact) or “Beyond BACI” sampling design^25^ was not possible in this study owing to the lack of pre-DSTP benthic community data. We used the geography and bathymetry of the Lihir and Misima study areas to overcome this handicap, selecting stations with differing levels of tailings input, and with the potentially confounding effects of water depth closely controlled. In this deep-sea setting, manipulative experiments generating direct cause-and-effect evidence^26^,^27^,^28^ are extremely difficult to perform, and impact assessment therefore rests on inference and correlation between infaunal community structure and sediment tailings content. Nevertheless, our results from an active and a closed mine are consistent with each other, with published data on coastal tailings disposal and from analogous large-scale sedimentation events in the deep sea, and it is reasonable to conclude that the observed patterns are attributable to the effects of DSTP.

Our results demonstrate clearly that ongoing DSTP at Lihir is associated with greatly reduced infaunal abundance and changes in higher-taxon composition. The scale of impact on metazoan meiofauna and calcareous forams declines with depth (and thus, distance from the tailings outfall) but is still significant down to 1700 m. Macrofauna and organic-walled forams are severely impacted to at least 2000 m. These patterns are consistent with published studies reporting substantial loss (or disappearance) of benthic forams^29^, metazoan meiofauna^30^ and macrofauna^31^,^32^,^33^ in coastal sediments exposed to mine tailings deposition. Deep-sea analogues also support this interpretation of the Lihir results. In the Cassidaigne Canyon (French Mediterranean slope) used for long-term disposal of aluminium smelting waste (“red mud”), meio- and macrofaunal densities were much lower at stations in the main canyon axis compared with peripheral stations receiving lower sediment input^34^. In the South China Sea, deposition of 6–8 cm of ash from the 1991 Mount Pinatubo eruption resulted in mass mortality of benthic forams^35^. Physical smothering is considered to drive the loss of both macrofauna^31^,^32^,^33^,^34^ and benthic forams^29^,^35^, whose upward mobility is severely curtailed by superficial deposits >2 cm in thickness^36^. Nevertheless, the thick surface tailings layer at stations L1-L3 was not completely azoic, some metazoan meiofauna always being present. In shallow-water experiments, harpacticoid copepods began to recolonise defaunated tailings in as little as 40 days, with numbers returning to background levels after 97–203 days^28^. Nematode recolonisation was slower, possibly reflecting the lack of a dispersive larval stage in this phylum. The copepod-dominated meiofauna found at the impacted Lihir stations may therefore be maintained by a continuous input of drifting propagules onto the freshly-deposited tailings.

The effects of DSTP at Lihir are detectable up to ~20 km east of the discharge point and to at least 2000 m water depth, but the full spatial and bathymetric extent of impact remains to be determined by a broader-scale survey. Our results mark the first essential step in mapping the benthic ecological “footprint” of the Lihir mine and for monitoring changes in its extent as tailings discharge continues.

At Misima, metazoan meiofauna, macrofauna and benthic forams all showed a clear contrast between stations with high (M1-M3) and low or no (M4-M6) tailings content. Data for the latter two infaunal groups suggest some recovery in total abundance, but with persistent effects on community structure. Benthic foram communities exposed to mine tailings^29^ and volcanic ashfall^35^ show very rapid (<1 year) return to background abundance once sediment deposition has ceased, but remain at low diversity and characterised by a few opportunistic taxa for up to 10 years^29^. In the Cap Breton Canyon (Bay of Biscay), foram assemblages 6–9 months after turbidite deposition were dominated by species rare or absent in undisturbed open slope sediments^37^. Low diversity and dominance by two species also characterised foram communities close to the “red mud” outfall in the Cassidaigne Canyon^38^. In such physically unstable environments the early recolonisation state may persist more or less indefinitely^37^. Data from coastal^26^,^31^,^32^ and deep-sea^34^ case studies also suggest recovery of macrofaunal abundance and species richness within three years after the end of tailings deposition, but with sediment instability again having a confounding effect. In a Canadian fjord used for tailings disposal, macrofaunal recovery was disrupted by slope failures and resuspension events, the impacts of which could equal or exceed those of the original tailings deposition^39^. Recovery rates may also be taxon-specific, with amphipods, for example, reported to be highly sensitive to sediment instability^32^. Foraminiferan and macrofaunal data from stations M1-M3 3.5 years post-DSTP are therefore consistent with a degree of community recovery (from an impacted state resembling the active DSTP stations east of Lihir), but with the successional process slowed or interrupted by physical disturbance. In this seismically-active region, periodic slumps of accumulated sediment down the steep Bwagaoia Basin slope would be expected, and are likely to have generated the disturbed bedforms observed at M1.

Meta-analysis of published case studies shows that trace metals (and other classes of contaminants) reduce the richness and evenness of marine communities^40^. However, without controlled experiments it can be difficult to separate the effects of chemical toxicity and physical instability in contaminated sediments^41^. The sensitivity of metazoan meiofauna to porewater copper has been observed in the field^30^ and confirmed by laboratory bioassays^27^,^42^ where abundance and diversity were significantly reduced above a threshold of 50 μg Cu L^−1^. Porewater copper concentrations at M1, where metazoan meiofauna were extremely sparse, were substantially above this threshold across most of the upper ~4 cm of the sediment column and consistently higher than at stations M3, M4 and M6 (Supplementary Fig. S1a). Values for cadmium, another ecotoxic trace metal, were also much higher at Bwagaoia Basin stations M1 and M3 than outside; lead showed no consistent difference, while dissolved arsenic levels were highest at the low/no tailings stations M4 and M6 (Supplementary Fig. S1b-d). With respect to macrofaunal recovery in tailings-affected sediments, residual contaminants have generally been considered less important than physical stability^26^,^31^,^32^,^39^. However, 15 years after the end of tailings discharge at the Black Angel mine (Greenland), dominance by opportunistic species at stations above a threshold value (200 mg kg sediment^−1^) for solid-phase lead was interpreted as evidence for a persistent trace metal contaminant effect^43^. Results of coastal field studies or laboratory bioassays must be applied with caution to the very different environment of Misima, but they suggest that tailings-derived contaminants may compound the effects of physical disturbance on rates of community recovery in the Bwagaoia Basin depocentre.

For all infaunal taxa sampled, station M4, where geochemical evidence indicated some tailings input, grouped with the tailings-free stations M5 and M6 rather than with the higher-tailings stations in the Bwagaoia Basin (M1-M3). This suggests the possible existence of a threshold level of tailings input required to drive detectable changes in infaunal communities. Future work should aim to test this hypothesis and identify any key sediment parameters involved (e.g. particulate deposition or ecotoxic metal content).

The level of taxonomic resolution needed to detect environmental impacts has been much debated, with recent literature focusing on the application of “Taxonomic Sufficiency” (TS)^44^ (identification to higher-taxon level only) to benthic faunal samples. Several coastal and shelf studies support community analysis at Family, Order or Class level^45^,^46^,^47^, while others caution against the potential loss of information in comparison with full species-level identification^48^,^49^. In the deep sea, species-level identification is often difficult and the environmental tolerances of individual species largely unknown, making the efficacy (or otherwise) of TS a particularly important issue^50^. Our results show that significant effects of DSTP are apparent at Family (Polychaeta) or higher-taxon level (Phylum, Class or Order for other faunal components). The benthic “footprint” of the *Deepwater Horizon* oil spill has recently been mapped from samples analysed at a similar level of taxonomic resolution^51^, suggesting that TS may be an effective tool for detection of large-scale pollutant impacts in the deep sea.

With interest in commercial seabed mining growing rapidly^52^,^53^, and continuing use of DSTP in developing nations, a better understanding of the impacts of large-scale anthropogenic disturbance on deep-sea benthic ecosystems is an essential step towards effective stewardship of these environments^54^,^55^. Our results show that significant community effects of DSTP are apparent even at a coarse level of taxonomic resolution and provide the basis for future monitoring of recovery rates in impacted deep-sea sediments.

## Methods

### Sediment collection and seabed photography

Sampling was carried out between 10 November and 5 December 2007 from the MV *Miss Rankin*. Sediments were collected using a hydraulically-damped multiple corer taking a maximum of eight cores (internal diameter 10 cm) drop^−1^. Cores showing evidence of slippage or disturbance were discarded. Seabed photographs were taken using a 35 mm film camera and strobe light in separate pressure housings, mounted in a steel frame and deployed vertically by wire from the vessel. An image was taken each time a weight suspended below the frame made contact with the seabed. Available time allowed drop-camera deployments at four stations (L1, L4, M1, M5).

### Sediment geochemistry

Cores required for geochemical analysis were extruded, sectioned and processed under N_2_. Cores were sectioned at 0.5 cm intervals to 5 cm depth, then at 1 cm intervals to 20 cm. Pore waters were separated by centrifugation at 4000 rpm and filtered through 5 μm then 0.2 μm pore size. Solid-phase samples were treated with HF in a microwave digester to achieve total sediment dissolution and the resulting solutions analysed by Inductively Coupled Plasma Mass Spectrometry (ICP-MS) and Inductively Coupled Optical Emission Spectrometry (ICP-OES). Pore water samples were diluted with 5% nitric acid and analysed for trace metals using ICP-MS.

### Benthic faunal analysis

Biological samples were collected from 3–7 replicate corer drops at each station (time constraints and equipment malfunctions prevented us from achieving the desired uniform level of sample replication at all stations). Cores required for faunal analysis were extruded on a vertical stand and sectioned at 5 cm and 10 cm depth horizons. The 0–5 cm and 5–10 cm layers were fixed separately in buffered 4% formaldehyde solution with Rose Bengal stain. Overlying water or unconsolidated surface sediment was removed by pipette before core sectioning and added to the 0–5 cm sample. In the laboratory, sediments were washed through a 250 μm sieve and residues transferred to 70% alcohol. Retained material was sorted under binocular microscope and metazoans counted and identified to higher-taxon (Phylum, Class or Order) level. All individuals of Nematoda, Ostracoda and Copepoda were classed as meiofauna. This taxonomic definition is unambiguous, reflects common ecological characters of the taxa concerned and is widely-used in the deep-sea literature in preference to the “traditional” definition using sieve mesh categories. In all samples the vast majority of metazoan meiofaunal individuals were present in the 250 μm sieve fraction. Benthic Foraminifera (forams) were not a principal focus of this study but stained (i.e. alive when collected) individuals in the >250 μm size fraction were counted and identified to major taxon (Class or Order) and morphotype (organic-walled or calcareous). Data were not collected on agglutinated forams owing to the difficulty of quantifying these easily-fragmented taxa. Data for metazoan meiofauna and benthic forams refer to the 0–5 cm sediment layer. Macrofaunal data refer to the pooled 0–5 and 5–10 cm layers (the vast majority of individuals were found in the 0–5 cm layer). All faunal densities were standardised to individuals m^−2^, with separate corer drops treated as replicate samples. Small (and sometimes uneven) sample sizes, and departures from assumptions of normality and homogeneity of sample variance made the use of parametric statistics inappropriate for these data. The nonparametric Mann-Whitney U-test^56^ was therefore used for the key comparisons between sampling stations with differing levels of tailings input. Among the macrofauna, polychaete worms were further identified to Family level. Polychaete community composition around Misima was compared by hierarchical cluster analysis using Primer^TM^ v.6^24^. Family-level abundance data for replicate corer drops at each station were standardised to ind. m^−2^ to correct for small differences in the number of individual cores per drop.

## Author Contributions

DJH was responsible for biological sampling, laboratory processing of samples and analysis of benthic community data. TMS was responsible for geochemical sampling, supervision of laboratory sample analysis and data processing. KDB and JAH were responsible for bathymetric survey and site selection and also contributed to sample collection at sea. All authors contributed to manuscript writing and editing.

## Additional Information

**How to cite this article**: Hughes, D. J. *et al.* Ecological impacts of large-scale disposal of mining waste in the deep sea. *Sci. Rep.* 5, 9985; doi: 10.1038/srep09985 (2015).

## Supplementary Material

Supporting Information Supplementary Figures 1 and Supplementary Tables 1-6

## Figures and Tables

**Figure 1:**
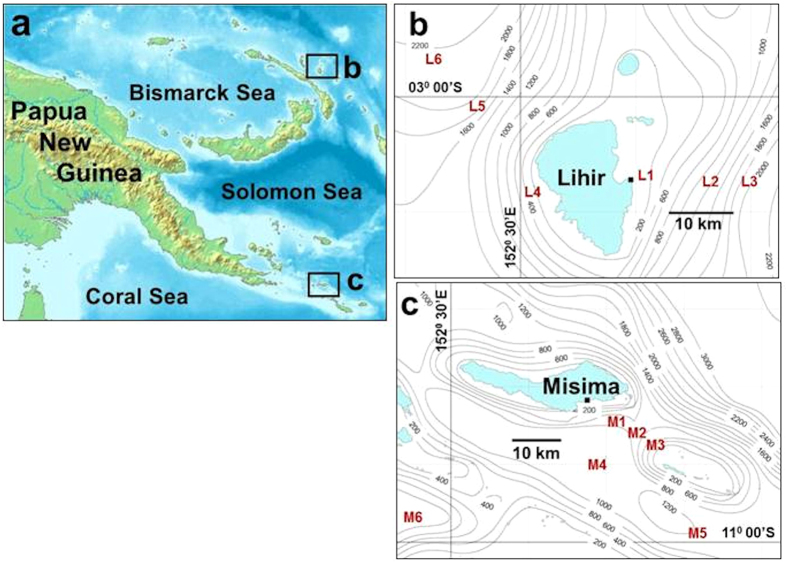
Study areas, Papua New Guinea (**a**) Locations of Lihir and Misima islands. Map modified from original on Wikimedia Commons, source www.demis.nl. (**b**) Benthic sampling stations off Lihir (L1-L6) and (**c**) Misima (M1-M6). Tailings outfalls are indicated by solid squares on island coastlines. Detailed bathymetric charts are not available for the Lihir and Misima areas, and (**b**) and (**c**) show only general trends using data from the General Bathymetric Chart of the Oceans (GEBCO, www.gebco.net).

**Table 1:** Abundance of living (Rose Bengal-stained) benthic Foraminifera (>250 μm) at stations east (L1-L3) and west (L4-L6) of Lihir.

Station (depth, m)	Organic-walled (Allogromiida)	Calcareous
L1 (850 m)	-	-
L2 (1750 m)	-	-
L3 (2020 m)	-	297 ± 321
L4 (800 m)	382 ± 459	1146 ± 441
L5 (1715 m)	425 ± 294	2504 ± 1396
L6 (2020 m)	297 ± 194	297 ± 265

Data show mean (± SD) number of individuals m^−2^ in the 0–5 cm depth horizon of replicate cores (n = 3) from each station, with replicates taken from separate corer drops.

**Table 2:** Abundance of living (Rose Bengal-stained) benthic Foraminifera (>250 μm) at stations around Misima.

Station (depth, m)	Organic-walled (Allogromiida)	cf. *Quinqueloculina* sp.	cf. *Buliminella* sp.	Other calcareous taxa
M1 (1380 m)	127 ± 128	4753 ± 1141	16976 ± 8571	1401 ± 459
M2 (1461 m)	382 ± 337	6791 ± 3808	12944 ± 3321	1485 ± 368
M3 (1467 m)	212 ± 265	5984 ± 2085	-	340 ± 389
M4 (1793 m)	594 ± 448	-	42 ± 73	1783 ± 764
M5 (1704 m)	849 ± 447	-	-	6026 ± 1696
M6 (1250 m)	2419 ± 1215	-	-	5560 ± 1141

Data show mean ( ± SD) individuals m^−2^ in the 0-5 cm depth horizon of replicate cores (n = 3) from each station, with replicates taken from separate corer drops.
